# Rapid Detection of *PML::RARA* Fusions in Acute Promyelocytic Leukemia: CRISPR/Cas9 Nanopore Sequencing with Adaptive Sampling

**DOI:** 10.3390/biom14121595

**Published:** 2024-12-13

**Authors:** William Middlezong, Victoria Stinnett, Michael Phan, Brian Phan, Laura Morsberger, Melanie Klausner, Jen Ghabrial, Natalie DeMetrick, Jing Zhu, Trisha James, Aparna Pallavajjala, Christopher D. Gocke, Maria R. Baer, Ying S. Zou

**Affiliations:** 1 Krieger School of Arts and Sciences, Johns Hopkins University, Baltimore, MD 21218, USA; wmiddle2@jhu.edu (W.M.); mphan4@jhu.edu (M.P.); 2 Department of Pathology, Johns Hopkins University School of Medicine, Baltimore, MD 21287, USA; vlloyd3@jhmi.edu (V.S.); lmorsber@jhmi.edu (L.M.); mhardy22@jhmi.edu (M.K.); jghabri1@jh.edu (J.G.); nescola1@jhu.edu (N.D.); jzhu23@jh.edu (J.Z.); tjames17@jhmi.edu (T.J.); apallav2@jhmi.edu (A.P.); cgocke1@jhmi.edu (C.D.G.); 3 Department of Biology, The College of William and Mary, Williamsburg, VA 23186, USA; bphan3@jh.edu; 4 Department of Medicine, University of Maryland Greenebaum Comprehensive Cancer Center, Baltimore, MD 21201, USA; mbaer@umm.edu

**Keywords:** CRISPR/Cas9, nanopore sequencing, *PML::RARA* fusions, acute promyelocytic leukemia, adaptive sampling

## Abstract

Acute promyelocytic leukemia (APL) accounts for approximately 10–15% of newly diagnosed acute myeloid leukemia cases and presents with coagulopathy and bleeding. Prompt diagnosis and treatment are required to minimize early mortality in APL as initiation of all-trans retinoic acid therapy rapidly reverses coagulopathy. The *PML::RARA* fusion is a hallmark of APL and its rapid identification is essential for rapid initiation of specific treatment to prevent early deaths from coagulopathy and bleeding and optimize patient outcomes. Given limitations and long turnaround time of current gene fusion diagnostic strategies, we have developed a novel amplification-free nanopore sequencing-based approach with low cost, easy setup, and fast turnaround time. We termed the approach CRISPR/Cas9-enriched nanopore sequencing with adaptive sampling (CENAS). Using CENAS, we successfully sequenced breakpoints of typical and atypical *PML::RARA* fusions in APL patients. Compared with the standard-of-care genetic diagnostic tests, CENAS achieved good concordance in detecting *PML::RARA* fusions in this study. CENAS allowed for the identification of sequence information of fusion breakpoints involved in typical and atypical *PML::RARA* fusions and identified additional genes (*ANKFN1* and *JOSD1*) and genomic regions (13q14.13) involving the atypical fusions. To the best of our knowledge, involvements of the *ANKFN1* gene, the *JOSD1* gene, and the 13q14.13 genomic region flanking with the *SIAH3* and *ZC3H13* genes have not been reported in the atypical *PML::RARA* fusions. CENAS has great potential to develop as a point-of-care test enabling immediate, low-cost bedside diagnosis of APL patients with a *PML::RARA* fusion. Given the early death rate in APL patients still reaches 15%, and ~10% of APL patients are resistant to initial therapy or prone to relapse, further sequencing studies of typical and atypical *PML::RARA* fusion might shed light on the pathophysiology of the disease and its responsiveness to treatment. Understanding the involvement of additional genes and positional effects related to the *PML* and *RARA* genes could shed light on their role in APL and may aid in the development of novel targeted therapies.

## 1. Introduction

Acute promyelocytic leukemia (APL) accounts for approximately 10–15% of newly diagnosed acute myeloid leukemia cases and presents with coagulopathy and bleeding [1]. Prompt diagnosis and treatment are required to minimize early mortality in APL as initiation of all-trans retinoic acid (ATRA) therapy rapidly reverses coagulopathy [2]. APL is characterized by t(15;17)(q24;q21), a balanced reciprocal translocation between the promyelocytic leukemia (*PML*) gene on chromosome 15 and the retinoic acid receptor alpha (*RARA*) gene on chromosome 17, leading to *PML::RARA* fusion [1]. This *PML::*RARA fusion protein disrupts normal myeloid differentiation and maturation, alters gene expression, deregulates transcriptional control (acting as a transcriptional repressor of *RARA* target genes), and disrupts PML homeostatic function [1]. It plays a critical role in the development of APL. Various breakpoints of *PML::RARA* fusion have been described. Given that *RARA* breakpoints are commonly located in intron 2, typical *PML::RARA* fusion transcripts depend on the *PML* breakpoints located in intron 6, exon 6, or intron 3, also known as bcr1 to bcr3 isoforms, respectively. Although rare, atypical *PML::RARA* fusion transcripts involving other regions of *PML* and *RARA* (such as exons 1 and 3 of the *RARA* gene and exons 4 and 7a–c of the *PML* gene), insertions of *PML::RARA* fusions, three-way translocations, variant complex translocations, and complex rearrangement have also been described [3,4,5,6,7,8]. The *PML::RARA* fusion is a hallmark of APL and its rapid identification is essential for rapid initiation of specific treatment to prevent early deaths from coagulopathy and bleeding and optimize patient outcomes.

Current gene fusion diagnostic strategies in the clinical setting include conventional karyotyping, fluorescent in situ hybridization (FISH), real-time quantitative reverse transcription polymerase chain reaction (qRT-PCR), and next-generation sequencing (NGS) assays. All these strategies have limitations and turnaround times ranging from days to weeks. While conventional karyotyping usually has a long turnaround time (~1 week), in-house rush/“STAT” FISH is commonly used to detect *PML::RARA* gene fusions within a day. These two standard-of-care cytogenetic methods cannot provide fusion breakpoint sequencing information. qRT-PCR is capable of detecting typical *PML::RARA* transcripts, but it generally fails to detect atypical transcripts involving variant *PML::RARA* fusion breakpoints, insertions of genomic DNA sequences at the junction of the *PML* and *RARA* genes, *PML::RARA* fusions, or partial deletions of the *RARA* or *PML* genes [3,4,5,6]. qRT-PCR requires high-quality RNA of adequate length and may exhibit strand bias due to PCR amplification process. Given that atypical *PML::RARA* fusions may be undetectable after standard amplification by routine qRT-PCR, direct sequencing or personalized patient-specific qRT-PCR is usually required to identify atypical breakpoints within the *PML* and *RARA* genes [4,5]. Recently, NGS-based methods have revealed reliable gene fusion data. However, they require high-complexity laboratories, are costly to set up, and are usually performed on batched specimens or sent out to centralized laboratories, leading to a longer turnaround time.

The advances in long-read sequencing allowed us to address these limitations. Nanopore sequencing is one of the long-read sequencing approaches. It functions based on a principle akin to that of a Coulter counter, where the electrical current passing through a small aperture is measured [9]. In nanopore sequencing, the pore is on the nanometer scale in diameter, precisely sized to permit the passage of only a single-stranded DNA molecule at a time. As a DNA molecule translocates through the pore, it partially obstructs the ion flow, leading to fluctuations in the ionic current. These changes are unique to the specific nucleotide bases (adenine, thymine, cytosine, and guanine) passing through the pore, enabling real-time identification of the DNA sequence. This method provides a direct, high-resolution readout of the DNA sequence, leveraging the distinct current disruptions caused by each nucleotide base. This technology offers distinct advantages over traditional sequencing methods, especially in detecting large structural variants (SVs). Long-read nanopore sequencing shows exceptional potential for characterizing chromosomal structural abnormalities, such as reciprocal translocations that result in fusion genes, like *PML::RARA* fusions in APL patients. Given the capability of sequencing stretches of DNA of up to a few kilobases in length, long-read nanopore sequencing enables the identification of breakpoints in structural abnormalities and uncovers genes or genomic regions involved in potential gene fusions or rearrangements caused by these structural changes. It offers a direct insight into large SVs and is capable of identifying and characterizing complex structural rearrangements (e.g., large deletions, duplication/amplification, insertions, translocations, and chromoanagenesis), without the need for additional specialized assays. Unlike other sequencing methods that require the amplification of DNA fragments before sequencing, nanopore sequencing allows for the direct reading of native DNA strands without requiring PCR-based amplification. It eliminates PCR amplification biases and enables the sequencing of complex regions or SVs, which are frequently challenging for traditional methods. In contrast to traditional sequencing methods where sequencing data are generated after extensive laboratory processing and results can take days or even weeks, one of the standout features of nanopore sequencing is its ability to produce data in real time. This real-time capability significantly accelerates the diagnostic process, making it ideal for urgent clinical applications where time is critical, such as detecting a *PML::RARA* fusion in APL patients. By delivering immediate results of *PML::RARA* fusion, nanopore sequencing enables faster decision-making and timely interventions in APL patients. The ability of nanopore sequencing to detect large, complex SVs, its real-time direct DNA reading capability, and its absence of amplification bias make it an invaluable tool for advancing modern genomics and clinical diagnostics.

Furthermore, the nanopore sequencer presents a highly viable and accessible tool for clinical testing. Unlike other sequencing technologies, which require significant capital investment, a considerable physical footprint, and the need for specialized technical expertise, nanopore sequencing provides a more cost-effective and user-friendly alternative. The MinION sequencing device, about the size of a large USB stick, has a low capital cost (around $1000) and is easy to operate (Oxford Nanopore Technologies (ONT), Oxford, UK). These features make the MinION a valuable and increasingly practical tool for clinical diagnostics. Many traditional sequencing methods, particularly those relying on large-scale sequencers, necessitate sophisticated laboratory infrastructure and highly skilled personnel, limiting their availability in low-resource or remote settings. In contrast, nanopore sequencing can be conducted using portable devices including the MinION, and PromethION 2 sequencers, which are compact, lightweight, and designed to operate in less specialized environments. This portability empowers genomic diagnostics in diverse clinical settings, including mobile health facilities and remote or rural healthcare centers, thereby enhancing access to diagnostic tools for structural abnormalities, such as the *PML::RARA* fusion in APL patients, worldwide. Moreover, the affordable nanopore flow cells for MinION and Flongle (~$80, ONT, Oxford, UK) further enhance the accessibility of nanopore sequencing, promising to have a significant impact on the clinical diagnostics field [10,11]. Nanopore technology offers a relatively low cost per sample, making it an attractive option for rapid diagnostics and the detection of structural abnormalities, such as the *PML::RARA* fusion in APL patients, across a wide range of patient populations. As technology evolves, nanopore sequencing has the potential to revolutionize the detection and diagnosis of SVs, providing fast, accurate, and more accessible diagnostic solutions.

Adaptive sampling in nanopore sequencing is an advanced technique to monitor and control individual nanopore in real time and to select specific sequence reads via real-time data analysis during nanopore sequencing [12,13,14,15,16]. The adaptive sampling approach allows us to enrich specific genomic regions and genes of interest via real-time data analysis (base-calling). Based on its enrichment mode, implemented using a simple Browser Extensible Data (BED) or FASTA file contains the target sequence regions or genes of interest. It can also deplete sequence reads from unwanted genomic regions during nanopore sequencing by utilizing real-time base-calling and voltage reversal at specific pores to reject molecules that are not of interest. The adaptive sampling approach has successfully characterized depletion and enrichment performance for DNA nanopore sequencing and complex transcriptome subsets by direct RNA nanopore sequencing [17]. Given real-time data analysis that adaptively targets specific genomic regions or genes of interest (with relevant sequences), adaptive sampling improves the run speed and efficiency of sequencing. This makes it a valuable approach to facilitate the use of nanopore sequencing in research and clinical diagnostic fields.

Clustered, regularly interspaced short palindromic repeats (CRISPR)-associated Cas9 nuclease system, an adaptive bacterial immune system that can cleave DNA, enables programmable targeting at a specific genomic DNA site [18,19,20,21]. CRISPR/Cas 9 technology has revolutionized the field of gene editing. It is a powerful genome-engineering tool for a wide range of applications in biomedical research and medicine [22,23,24,25,26,27,28]. Given its capability of creating targeted double-strand breaks, CRISPR/Cas9 has been successfully used to investigate gene function, increase our knowledge of human disease etiology through CRISPR-based disease models, and improve the treatment of human diseases through gene therapy and genome editing [25,29,30,31]. Recently, CRISPR/Cas9 technology has also been employed as a targeted enrichment strategy to focus on specific genomic regions, genes, or chromosomal DNA, utilizing adapter ligation for nanopore sequencing [32,33]. For this strategy, the guide ribonucleic acid (guideRNA) flanks specific genomic regions or genes and is custom designed. After the guideRNA aids in recognizing specific sequences of specific genomic regions or genes, the ends of Cas9 cut sites ligate to adaptors. This strategy allows for sequencing enriched regions and genes of interest to provide high sequencing depth and comprehensive variant detection without the loss of native modifications (such as CpG methylation).

We developed a novel nanopore sequencing-based approach for rapid detection of *PML::RARA* fusions in APL patients. We used CRISPR/Cas9 for targeted enrichment of the regions of interest with no PCR amplification steps before nanopore sequencing [32,33]. During nanopore sequencing, we used adaptive sampling for real-time sequencing [16]. Only nanopore sequencing data associated with the *PML* and *RARA* genes (defined as regions of interest) continued to be sequenced and collected for data analyses. We termed the approach CRISPR/Cas9-enriched nanopore sequencing with adaptive sampling (CENAS). An amplification-free sequencing approach has the advantages of low-cost investment, low cost per sample, and a fast turnaround time. It also offers long reads and real-time sequencing data generation using a palm-sized portable nanopore MinION sequencing system (Oxford Nanopore Technologies, Oxford, UK). Therefore, CENAS could develop as a point-of-care test that enables quick, accessible, and cost-efficient bedside diagnosis of APL patients with a *PML::RARA* fusion.

## 2. Materials and Methods

### 2.1. Cell Lines and Patient Specimens

The NB4 cell line was obtained from Accegen (Fairfield, NJ, USA) and the GM12878 cell line from Coriell Institute (Camden, NJ, USA). This study also included eighteen peripheral blood or bone marrow specimens from patients referred to the Johns Hopkins Hospital from 1 January 2022, to 30 June 2024. These patients had routine diagnostic procedures, including morphologic evaluation, flow cytometry, fluorescence in situ hybridization (FISH), conventional chromosome analysis, a real-time qRT-PCR, a gene fusion assay, a targeted next-generation sequencing (NGS) assay, and/or a single-nucleotide polymorphism (SNP) microarray analysis. Disease classification by standard pathology practice and delineated by the World Health Organization was based on clinical, morphologic, immunophenotypic, cytogenetic, and molecular genetic features. There were ten women and eight men, with a median age of 44 years (range, 20 to 65 years). Twelve specimens were APL patients at initial diagnosis and had a *PML::RARA* fusion by standard-of-care genetic diagnostic tests. Six specimens were patients with non-APL acute myeloid leukemia without a *PML::RARA* fusion by standard-of-care genetic diagnostic tests. This study was conducted after approval by the institutional review board (IRB) at the Johns Hopkins School of Medicine in accordance with the Declaration of Helsinki, the 1996 Health Insurance Portability and Accountability Act, and the ethical standards of the Institutional Committee on Human Experimentation (https://ictr.johnshopkins.edu/wp-content/uploads/2014/01/Overall-HSR-Section-FINAL-6-15-2017.pdf, accessed on 28 August 2024).

### 2.2. Cytogenetics Data: Conventional Chromosome Analysis, Fluorescence In Situ Hybridization (FISH), and SNP Microarray

Conventional G-banded chromosome studies were performed using standard techniques. A minimum of 20 metaphase cells were analyzed from bone marrow or peripheral blood specimens. The abnormal karyotypes were described using the International System for Human Cytogenetic Nomenclature (2020) [34].

FISH was performed on interphase nuclei using disease-specific panels of probes according to the manufacturer’s protocol and following the previously published protocol [35]. The FISH panel included a *RARA* (17q) break-apart probe set and a dual color dual fusion *PML/RARA* probe set (Abbott Molecular, Inc., Des Plaines, Cook, IL, USA). For each probe, 200 nuclei were visually evaluated with fluorescence microscopy by two technologists scoring separately using a Zeiss Axioscope system (Carl Zeiss Microscopy, LLC, Oberkochen, Germany), blinded from each other. The analysis was performed using Cytovision software version 7.7. The specimen was considered abnormal if the results exceeded the laboratory-established cutoff for each probe set.

Whole-genome SNP microarray analysis was performed according to the manufacturer’s protocol and following the previously published protocol [33]. DNA was extracted from bone marrow or blood specimens by conventional methods (Qiacube). The DNA concentration was assessed using a Qubit fluorometer, using the DNA broad range (BR) reagent (Thermo Fisher Scientific, Waltham, MA, USA). The high-resolution microarray platform utilized was the Illumina Infinium CytoSNP−850 K v1.2 BeadChip containing >850,000 markers (mean spacing, 3.5 kb; Illumina, Inc., San Diego, CA, USA). BeadChips were processed per manufacturer’s guidelines and imaged with the Illumina iScan system. Data were analyzed with the CNV Partition 2.4.4.0 algorithm in GenomeStudio version 2010.3 (Illumina) and KaryoStudio version 1.4.3.0 (Illumina). B allele frequency and logR signal intensities were used to examine and identify potential pathogenic regions of genomic imbalance. All analyses were performed using human reference genome assembly hg19 (GRCh37).

### 2.3. CRISPR/Cas9-Mediated and Adaptive Sampling by Nanopore Sequencing

For the NB4 and GM12878 cell lines, DNA was extracted with MasterPure (VWR, Radnor, PA, USA) following a standard protocol. For patient samples, DNA was extracted with PureGene (Qiagen, Germantown, MD, USA) following a standard protocol. Bone marrow samples typically contain a higher concentration of white blood cells than whole blood samples. After adding the cell lysis solution, we ensured the mixture was thoroughly homogeneous. If the solution remained uneven, we added more of the cell lysis solution and proportionally increased the volumes of the other reagents used in the protocol to maintain the proper balance. Three micrograms of DNA were used to generate the sequencing library. crRNA guides were designed using Custom Alt-R CRISPR-Cas9 guide RNA (https://www.idtdna.com/site/order/designtool/index/ CRISPR_ CUSTOM, accessed on 1 February 2023) and Chopchop (https://chopchop.cbu.uib.no/, accessed on 1 February 2023) with CRISPR-Cas9. crRNA guides were designed to direct Cas9 to cut on introns 2, 3, 4 and exons 3, 4, and 5 of RARA and introns 2, 3, 5, and 7 of PML and were obtained from Integrated DNA Technologies (IDT, Inc., Coralville, IA, USA).

CRISPR/Cas9-guided targeted enrichment and adapter ligation followed the previously published protocol [32,33] with modifications. Briefly, after dephosphorylation of free DNA ends and cleavage and A-tailing of DNA, we added an ethanol wash step before the adapter ligation steps. The ethanol wash step improved pore occupancy by increasing the number of active pores performing sequencing, thereby shortening the sequencing runtime. For the ethanol wash step, we added 43 µL of re-suspended AMPure XP beads (AXP) (Beckman Coulter Life Sciences, Sykesville, MD, USA) to the end-prep reaction and incubated on a HulaMixer sample mixer (Thermo Fisher Scientific, Halethorpe, MD, USA) for 5 min at room temperature. Then, we kept the tube on the magnet, pipetted off the supernatant, and washed the beads two times with 200 µL of freshly prepared 80% ethanol without disturbing the pellet. We removed the ethanol, spun it down, placed the tube back on the magnet, pipetted off any residual ethanol, and allowed it to dry for 1 min. We removed the tube from the magnet and re-suspended the pellet in 43 µL nuclease-free water, incubated it for 4 min at room temperature, kept the pellet on a magnet for 1 min, and removed and retained 43 µL of eluate into a clean 1.5 mL Eppendorf DNA LoBind tube. After the ethanol wash step, we continued to go through the adapter ligation step, library preparation, and nanopore sequencing as previously published [32,33] using ligation sequencing kit V14 (SQK-LSK114, Oxford Nanopore Technologies (ONT), Oxford, UK). Briefly, we washed by adding AXP beads (Agencourt, Brea, CA, USA) at 0.5 × volume, and a long fragment buffer (ONT, Oxford, UK) was used for bead washes. Then, the library was eluted using an elution buffer (SQK- LSK114, ONT, Oxford, UK). The library was loaded on ONT flow cells (FLO-FLG114 R10 flongle flow cells or FLO-MIM114 R10 MinION flow cells) and sequenced on a nanopore sequencer (MinION or GridION sequencer, ONT, Oxford, UK). Since both the GridION and Mk1b with MinION used the same flow cell, and sequencing program and operated at a sequencing temperature of 37 °C or higher, no differences in QC or sequencing results were observed between the devices in this study. The only distinction is that the GridION sequencer can process up to five samples simultaneously, whereas the MinION Mk1b can handle only one sample at a time.

The base-calling algorithm was provided by Oxford Nanopore (ONT, Oxford, UK) and sequences were aligned to human genome builder GRCh37/hg19. The sequencing regions used for adaptive sampling were *PML* at chr15:74287014–74340155 and *RARA* at chr17:38465423–38566941 based on human genome builder GRCh37/hg19. SVs involving *PML* and *RARA* genes were reviewed independently by multiple genetic analysts via the Integrative Genomics Viewer (IGV, Broad Institute, Cambridge, MA, USA). The specimen was considered abnormal (positive for a fusion) if identical fusion breakpoints were revealed in more than two sequencing reads.

### 2.4. Real-Time Quantitative RT-PCR (qRT-PCR) Assays for PML::RARA Fusions

qRT-PCR assays were performed in a CLIA/CAP-certified molecular diagnostics lab. RNA was extracted from bone marrow or peripheral blood using standard procedures [Qiagen, Germantown, MD, USA]. RNA was reverse transcribed using high-capacity cDNA reverse transcription reagents (ABI), and fusion gene mRNA was detected by real-time PCR using the Qiagen *PML::RARA* Fusion Quant kits on a Taqman 7900 or QuantStudio 6 instrument. A control mRNA (ABL1) was amplified to monitor the quality of the sample. In acute promyelocytic leukemia (APL), the Qiagen Fusion Quant *PML::RARa* bcr 1 (L form), *PML::RARa* bcr2 (V form), and *PML::RARa* bcr3 (S form) assays detected and quantified a t(15;17) translocation.

### 2.5. RNA-Base Gene Fusion Assay for PML::RARA Fusions

The RNA-based gene fusion assay for *PML::RARA* fusions was performed using a comprehensive, NanoString technology–based targeted fusion panel. A total of 300 ng RNAs extracted from patient blood or bone marrow specimens was used for the assay, following the manufacturer’s protocol as previously described [36].

### 2.6. Next-Generation Sequencing (NGS) Assay

DNA was extracted from blood and bone marrow specimens by conventional methods (Qiacube; Qiagen, Hilden, Germany). DNA concentration was assessed by the Qubit fluorometer, using the DNA broad range (BR) reagent, according to vendor specification (Thermo Fisher Scientific, Halethorpe, MD, USA). The NGS assay has been described in previous studies [35,37]. Briefly, library preparation was performed using Kapa Roche Hyper Prep reagents (Roche Diagnostics, Inc., Wilmington, MA, USA); hybrid capture was executed using 40,670 Integrated DNA Technologies, Inc., probes and reagents, and products were sequenced using NovaSeq 6000 with NovaSeq Rapid Cluster and SBS v2 200-cycle reagents with Illumina paired-end technology (Illumina, Inc., San Diego, CA, USA). All analyses were performed using human reference genome assembly GRCh37/hg19. SVs were reviewed independently by multiple genetic analysts via the IGV (Broad Institute, Cambridge, MA, USA). Only clusters with at least three reads at each potential SV breakpoint were considered a putative SV junction.

### 2.7. Statistical Calculators

A comparison of concordance, including sensitivity, specificity, predictive values, and the accuracy, between CENAS and the FISH assay was conducted using MEDCALC statistical (MedCalc’s Diagnostic test evaluation calculator at https://www.medcalc.org/, last accessed 28 August 2024). Sensitivity (true positive rate), specificity (true negative rate), and predictive values were calculated from the confusion matrix for each assay. Accuracy was the proportion of correct predictions (both true positives and true negatives) out of all cases. McNemar’s test was used to assess the concordance between the two assays. The significance level was set at *p* < 0.05, which was considered statistically significant. No corrections were applied for multiple comparisons.

## 3. Results

**Figure 1:**
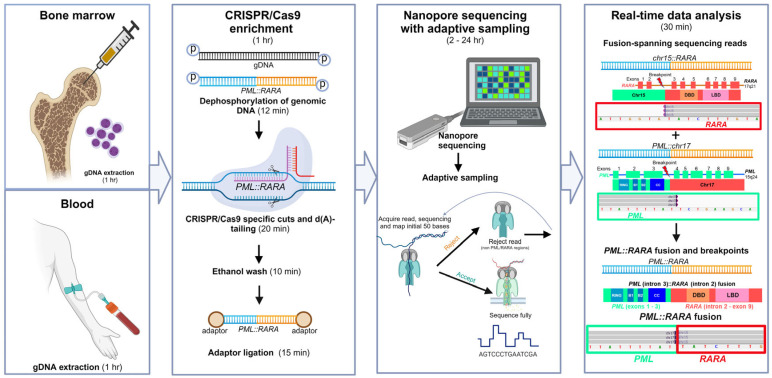
The CRISPR/Cas9-enriched nanopore sequencing with the adaptive sampling (CENAS) approach to reveal *PML::RARA* fusions in APL patients. The entire procedure includes DNA extraction from blood or marrow, CRISPR/Cas9-guided enrichment of targeted *PML* and *RARA* genomic regions, nanopore sequencing with adaptive sampling, and sequencing data analysis to reveal sequences involving *PML::RARA* fusions. hr: hours; min: minutes.

CENAS combined CRISPR/Cas9-targeted enrichment and the adaptive sampling approach to enrich the sequencing reads containing the *PML* and *RARA* genes and reduced sequencing runtime (Figure 1).

We used CRISPR/Cas9 for targeted enrichment of the regions of interest with no PCR amplification steps before nanopore sequencing. During nanopore sequencing, we used adaptive sampling for real-time sequencing. Only nanopore sequencing data associated with the *PML* and *RARA* genes (defined as regions of interest) continued to be sequenced and collected for data analyses. The CENAS approach optimized sequencing time and efficiency.

### 3.1. Summary of CENAS-Based Nanopore Sequencing Results

Using CENAS, we successfully sequenced the breakpoints of *PML::RARA* fusion in the APL cell line NB4 (Table 1, Supplemental Figure S1, and Supplemental Table S1). We performed CENAS on remnant DNA from eighteen blood or bone marrow specimens. Specimens included twelve APL specimens at initial diagnosis with *PML::RARA* fusion by standard-of-care genetic tests and six acute myeloid leukemia specimens without *PML::RARA* fusion (Table 1). Among the twelve APL specimens with *PML::RARA* fusion by dual color dual fusion FISH, eight specimens had the typical *PML::RARA* FISH signal pattern of a classic t(15;17) reciprocal translocation and four had an atypical FISH signal pattern (Cases #11–14 in Table 1). CENAS successfully detected *PML::RARA* fusions and characterized the breakpoint sequences in all twelve APL specimens carrying typical and atypical fusions (Table 1). As expected, CENAS did not detect any *PML::RARA* fusions in the six non-APL specimens (Cases #15–20 in Table 1) and no *PML::RARA* fusion reads were detected in non-APL GM12878 cells. Compared with the FISH test, CENAS achieved good concordance in detecting *PML::RARA* fusions in this study (Table 1).

The NGS assay was performed on APL cases #3, #6, #8, #9, #10, #12, #13, and non-APL cases #15, #16, #17, 19, and #20. The NGS assay successfully identified the breakpoint sequences associated with *PML::RARA* fusions in the APL specimens, with results that were consistent with those detected by CENAS. The potential fusion breakpoint sequences at the *PML::RARA* junction, identified by the NGS assay, were reviewed using IGV (Supplemental Figure S2). No potential *PML::RARA* fusions were observed in any of the non-APL specimens.

### 3.2. CENAS for Atypical and Cryptic PML::RARA Fusions

Four APL specimens had atypical *PML::RARA fusions* by dual color dual fusion FISH (Cases #11–14 in Table 1). They included three with a FISH signal pattern of only one single fusion due to cryptic insertions, three-way translocations, or complex rearrangements (Cases #11–13 in Table 1) and one with poly-fusions (case #14 in Table 1). CENAS was especially useful in detecting *PML::RARA* fusions due to complex rearrangements.

#### 3.2.1. Case #13: Atypical *PML::RARA* Fusion Due to Three-Way Translocation

**Figure 2:**
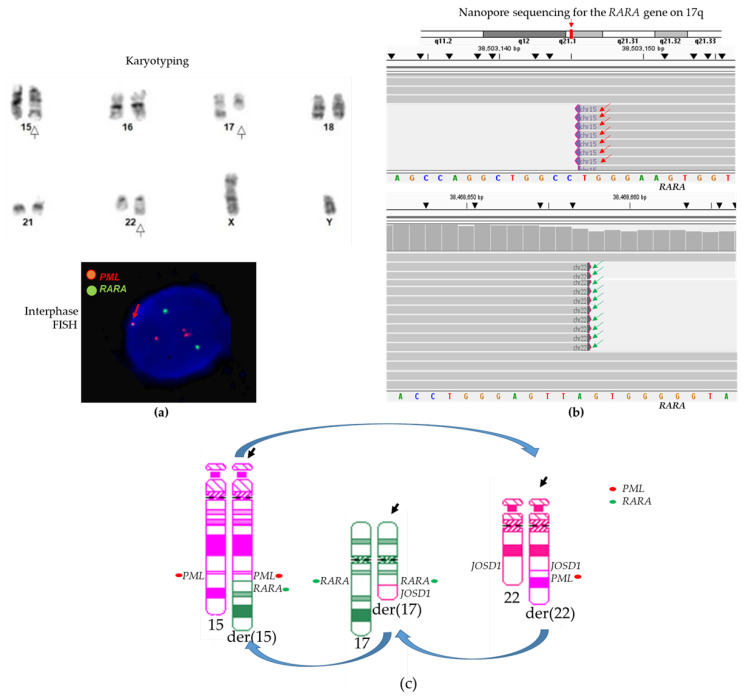
Atypical *PML::RARA* fusions in case #13. (**a**) Partial karyogram shows a t(15;22;17) three-way translocation (white arrows for abnormal derivative chromosomes). Below: Interphase FISH shows an atypical FISH signal pattern (2R2G1F). The red arrows point to a *PML::RARA* fusion. (**b**) CENAS shows sequence reads of a *PML::RARA* fusion involving the *PML* gene on chromosome 15q and the *RARA* gene on 17q (red arrows), and a *JOSD1::RARA* fusion involving the *RARA* gene on 17q and the *JOSD1* gene on chromosome 22 (green arrows). Sequences were aligned to human genome builder GRCh37/hg19. (**c**) Diagram of the t(15;22;17) three-way translocation and atypical *PML::RARA* fusion. Black arrows point to derivative chromosomes involved in this translocation.

Case #13 had a three-way translocation, t(15;22;17) by conventional chromosome analysis and an atypical *PML::RARA* fusion by FISH (Figure 2a). The sequencing-based CENAS approach detected fusions involving three genes (the *PML* gene on chromosome 15q, the *RARA* gene on chromosome 17q, and the *JOSD1* gene on 22q) from three different chromosomes 15, 17, and 22 (Figure 2b). A *PML::RARA* fusion was one of these fusions.

#### 3.2.2. Case #12: Cryptic and Atypical *PML::RARA* Fusion

**Figure 3:**
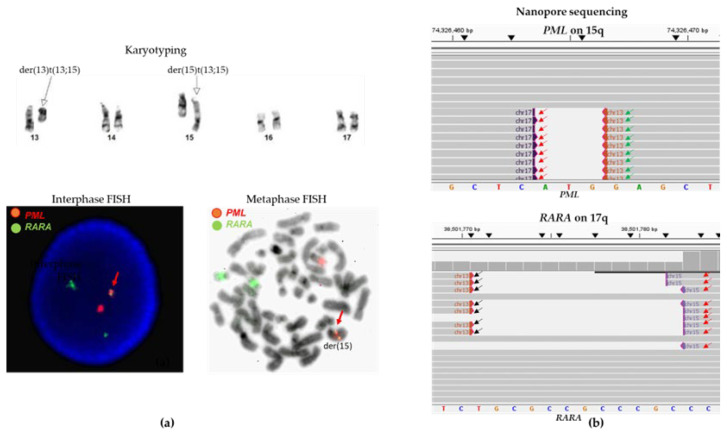
Cryptic *PML::RARA* fusion in case #12. (**a**) Partial karyogram shows a t(13;15) reciprocal translocation (white arrows for abnormal derivative chromosomes) with two normal chromosomes 17. Interphase FISH shows an atypical FISH signal pattern (1R2G1F). The red arrows point to a *PML::RARA* fusion. Metaphase FISH shows an insertional *PML::RARA* fusion into the derivative chromosome 15 formed by t(13;15) translocation, ish der(15)t(13;15)(q14;q24) ins(15;17)(q24;q21q21) (*PML* +, *RARA* +). (**b**) CENAS shows sequence reads of a complex atypical *PML::RARA* fusion involving chromosomes 13, 15, and 17. Red arrows point to a *PML::RARA* fusion/t(15;17) translocation, green arrows point to a fusion of the *PML* gene and 13q14.13 (chr13:46,515,010), and black arrows point to fusions of the *RARA* gene and 13q14.13 (chr13:46,515,891). These data support the presence of complex fusions and rearrangements. Sequences were aligned to human genome builder GRCh37/hg19.

Case #12 had a submicroscopic *PML::RARA* fusion, which was cryptic by conventional chromosome analysis and qRT-PCR. Given a t(13;15) reciprocal translocation by chromosome analysis and an atypical interphase FISH result, we performed metaphase FISH, revealing insertion of a *PML::RARA* fusion into the derivative chromosome 15 formed by the t(13;15) translocation (Figure 3a). With the presence of complex rearrangements involving various breakpoints, qRT-PCR was not sensitive enough to unequivocally detect this *PML::RARA* fusion. CENAS detected this *PML::RARA* fusion and revealed detailed sequencing data involving this case’s complex fusion and rearrangement, which also involved the genomic region of 13q14.13 (Figure 3b).

#### 3.2.3. Case #11: Atypical and Likely Insertional *PML::RARA* Fusion

**Figure 4:**
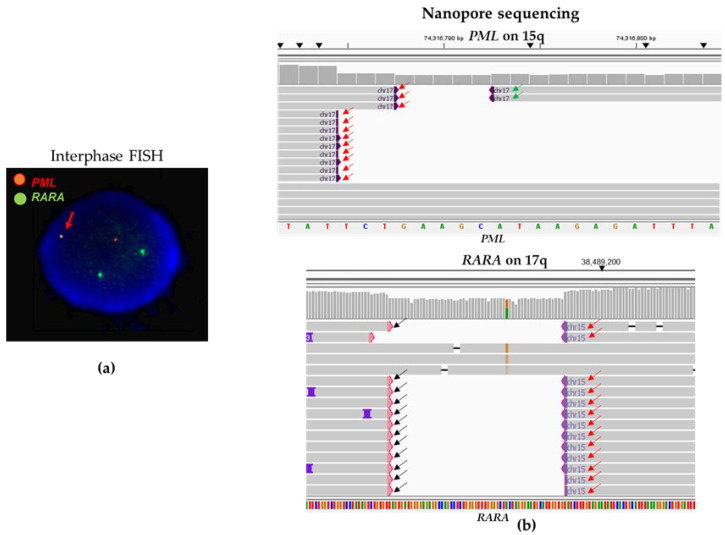
Atypical *PML::RARA* fusion in case #11. (**a**) Interphase FISH shows an atypical FISH signal pattern (1R2G1F). The red arrows point to a *PML::RARA* fusion. (**b**) CENAS reveals an atypical *PML::RARA* fusion (a likely insertional fusion). Red arrows point to a *PML::RARA* fusion, green arrows point to the *PML* gene fused to the *ANKFN1* gene on 17q22, and black arrows point to the *RARA* gene fused to the *ANKFN1* gene on 17q22. These data suggest the presence of a likely insertional *PML::RARA* fusion into a derivative chromosome 17. Sequences were aligned to human genome builder GRCh37/hg19.

Case #11 had an atypical *PML::RARA* fusion by interphase FISH (Figure 4a) and a normal SNP microarray result without any copy number variants (gains or losses) of the chromosomes 15 and 17, including the *PML* and the *RARA* genes. The sequencing-based CENAS approach detected fusions involving three genes (the *PML* gene on chromosome 15q, the *RARA* gene on chromosome 17q, and the *ANKFN1* gene on 17q22) from two chromosomes 15 and 17 (Figure 4b). Given the presence of a *PML::RARA* fusion and both *PML* and *RARA* genes fused to the *ANKFN1* gene on 17q22, CENAS sequence data suggest a likely insertional *PML::RARA* fusion.

#### 3.2.4. Case #14: Multiple *PML::RARA* Fusions by FISH

**Figure 5:**
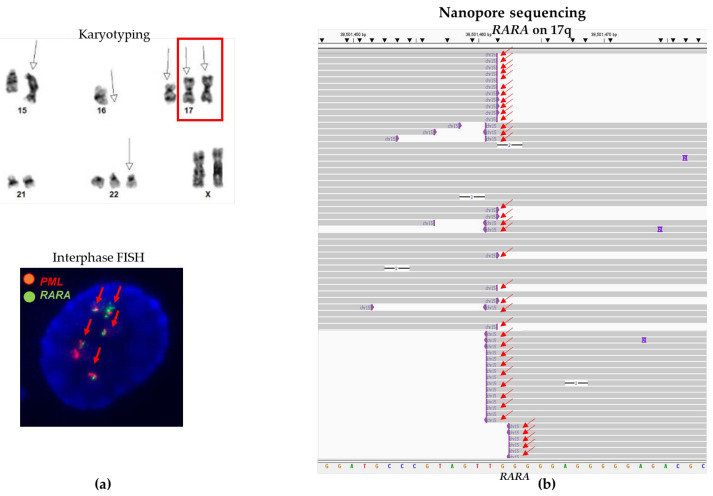
Multiple *PML::RARA* fusions in case #14. (**a**) Partial karyogram shows der(15), −16, +17, ider(17)(q10)t(15;17)x2, and +22 (white arrows for abnormalities). The red box shows isoderivative (ider) chromosome 17q involving t(15;17) translocations on both 17q arms. Interphase FISH reveals multiple fusions. (**b**) CENAS displays a higher number of sequencing fusion reads (indicated by red arrows) compared to non-fusion reads, due to the presence of multiple *RARA::PML* fusions in this case. Sequences were aligned to human genome builder GRCh37/hg19.

Case #14 had multiple (four to five) *PML::RARA* fusions due to concurrent isoderivative (ider) chromosome 17q arising from duplication of the ider(17q), with consequent overrepresentation of the reciprocal *RARA::PML* fusion and loss of 17p, including the tumor suppressor gene TP53 (Figure 5a). CENAS revealed *PML::RARA* fusions in this case (Figure 5b).

## 4. Discussion

In this study, CENAS successfully detected typical and atypical *PML::RARA* fusions in all APL patients studied. CENAS, a novel amplification-free nanopore sequencing-based approach with low cost and easy setup, has great potential for further development as a point-of-care test to offer an immediate bedside diagnosis of APL patients with a *PML::RARA* fusion, including atypical fusions. While classic/typical balanced t(15;17) rearrangements are found in most (~87%–~92%) APL cases, up to 13% of patients may have atypical and cryptic results by standard-of-care cytogenetic testing [38,39]. As a direct sequencing approach, CENAS has great potential to reveal typical, atypical, and cryptic *PML::RARA* fusions, some of which may be challenging to detect by standard-of-care cytogenetic tests and qRT-PCR. For atypical *PML::RARA* fusions in this study, CENAS not only detected and characterized breakpoints of *PML::RARA* fusions but also revealed additional genes/genomic regions involving additional fusions/rearrangements.

### 4.1. Pros and Cons of CENAS Compared with Other Emerging Diagnostic Methods

In this study, CENAS nanopore sequencing was performed using the latest R10.4.1 flow cell paired with the updated V14 chemistry. These advancements substantially improve read quality, enabling the generation of high-quality hybrid and long reads while also enhancing base-calling accuracy [40,41,42,43]. These improvements result from updates in base-calling algorithms, as well as refinements in library preparation and flow cell chemistry. Additionally, the R10.4.1 flow cell features a dual reader head design, which significantly enhances homopolymer sequencing accuracy [40,44]. Moreover, the V14 chemistry has greatly reduced error rates, achieving an accuracy of approximately 99% with the R10.4.1 flow cell [40,42,43], compared with around 94% with the previous V10 chemistry on the older R9.4.1 flow cell [45]. The combination of the R10.4.1 flow cell and V14 chemistry enhances the clinical applicability of nanopore sequencing for fusion detection. Given the advancements in other emerging diagnostic technologies, CENAS highlights its unique advantages as well as potential disadvantages.

#### 4.1.1. Comparison of CENAS Nanopore Sequencing with Pacific Biosciences’ Sequencing

For the past two decades, first- and second-generation short-read sequencing, typically producing reads of around 150 bp, has been the gold standard for genetic profiling. However, these technologies have limitations in detecting and characteriz-ing SVs, as well as in analyzing complex genomic regions, repetitive elements, or vari-ant phasing. Additionally, the PCR amplification used in sequencing templates can in-troduce artifacts and hinder the detection of native base modifications. In contrast, long-read sequencing, or third-generation sequencing, can produce sequence reads crossing kilobases or even megabases, which is extremely functional for detecting SVs. Long-read sequencing is also real-time and direct, PCR-free in both sequencing and li-brary preparation, effectively eliminating PCR-related biases. Single-molecule re-al-time sequencing from Pacific Biosciences and nanopore sequencing from ONT are the two leading technologies for long-read sequencing. Both companies are focused on enhancing the accuracy, throughput, and portability of their long-read sequencing methods while striving to keep costs low. Pacific Biosciences’ Revio system, utilizing HiFi reads, is based on the circular consensus sequencing model, enhanced by ad-vanced sequencing chemistry and deep learning algorithms. This combination results in an error rate comparable to that of short-read Illumina sequencing [46]. While na-nopore sequencing generally has higher error rates than Pacific Biosciences’ HiFi reads, recent advancements in flow cells, chemistry, and base-calling algorithms have signif-icantly improved its accuracy. These improvements make nanopore sequencing suffi-ciently reliable for many applications, including fusion detection. Both technologies are highly capable of fusion detection, but nanopore sequencing offers distinct advantages in terms of portability and flexibility, making it ideal for real-time, dynamic clinical applications. Nanopore sequencing platforms are typically more cost-effective, partic-ularly for smaller-scale patient populations, such as those with APL. In contrast, Pa-cific Biosciences’ systems are less portable, requiring more extensive laboratory infra-structure and a higher initial investment in equipment and consumables, making them better suited for large-scale sequencing projects. The MinION nanopore sequencer provides an affordable entry point for labs and clinicians seeking long-read sequencing for fusion detection without the burden of significant upfront costs. Moreover, na-nopore sequencing’s user-friendly workflow facilitates rapid sequencing, which is es-pecially valuable in clinical or point-of-care environments where timely results are critical for patients with conditions like APL.

#### 4.1.2. Comparison of CENAS with Other RNA-Based Gene Fusion Assays

Targeted RNA-based gene fusion assays, such as qRT-PCR, targeted RNA sequencing, and anchored multiplex PCR, are highly effective at detecting low-abundance transcripts [3,4,5,6,47,48]. These assays provide detailed and accurate profiles of gene fusions involving specific, predefined target genes. Most of these assays rely on PCR-based enrichment, making RNA integrity and proper sample preparation essential for ensuring accurate and reproducible results [47]. Additionally, most targeted assays are limited to detecting fusions within specific, predefined genes or regions, which means that novel or uncharacterized fusion events may be overlooked. In contrast, whole-transcriptome RNA sequencing (RNA-Seq) does not depend on predefined probes, allowing for the identification of novel genes, isoforms, and previously undiscovered fusion events. Despite this advantage, RNA-Seq generates large amounts of data, requiring significant computational resources and bioinformatics expertise for analysis. It also necessitates high-complexity laboratory setups, making it costly to implement. Typically performed on batched specimens or outsourced to centralized labs, RNA-Seq can result in longer turnaround times. While sequencing costs have decreased over time, RNA-Seq remains expensive, particularly for high-throughput or single-cell applications.

Direct-DNA nanopore sequencing-based CENAS can detect typical, atypical, and cryptic *PML::RARA* fusions, some of which could be difficult to identify using certain targeted RNA-based gene fusion assays. As an amplification-free approach, CENAS offers advantages such as lower cost, simpler setup, and faster turnaround time compared with RNA-Seq. These features position CENAS as a promising tool for point-of-care testing, potentially enabling immediate bedside diagnosis of APL patients with a *PML::RARA* fusion.

#### 4.1.3. Comparison of CENAS with Optical Genome Mapping

In addition to the standard cytogenetic methods, such as conventional karyotyping and FISH, which are routinely used to detect *PML::RARA* fusions in APL patients, optical genome mapping (OGM) is an emerging technology for characterizing genome-wide SVs. OGM provides a detailed view of DNA structure by generating long DNA molecules labeled with fluorescent tags at specific sequence motifs. These labeled molecules are then stretched and imaged, creating a map of the genome’s structure, including SVs like *PML::RARA* gene fusions caused by t(15;17) translocations. OGM is particularly useful for characterizing typical, atypical, and cryptic *PML::RARA* fusions in APL patients [49]. However, like conventional karyotyping and FISH, OGM cannot provide sequencing information about fusion breakpoints. Compared with OGM and conventional karyotyping (which takes about a week), CENAS offers a significantly faster turnaround time and is on par with in-house FISH assays.

### 4.2. Potential Clinical Impacts of Atypical PML::RARA Transcripts

Given different *PML* and *RARA* domains retained in the fusion protein, typical and atypical *PML::RARA* isoforms may be associated with diverse prognoses and treatment responsiveness. However, due to the rarity of t(15;17)-positive APL patients with atypical *PML::RARA* transcripts, the biological and clinical impact of these atypical isoforms is not fully understood. A few patients with atypical *PML::RARA* transcripts have had clinical outcomes similar to patients with typical fusion transcripts [5,50,51,52]. The V-type (for variable) *PML::RARA* transcripts found in approximately 8% of adult patients with APL are defined molecularly by truncation of *PML* exon 6 and frequent insertion of genetic material from *RARA* intron 2. V-type *PML::RARA* fusion transcripts in APL have shown decreased in vitro responsiveness to ARTA [53] and are reported to display decreased sensitivity to standard ARTA treatments [53,54] and a higher failure rate of standard treatments [55]. Furthermore, atypical *PML::RARA* transcripts, especially *PML* breakpoints at downstream intron 6, are potentially associated with aggressive disease and poor prognosis [4,56]. These transcripts may not respond to standard ATRA treatment [38]. CENAS characterized *PML::RARA* fusion breakpoints at a nucleotide level, which is extremely useful for determining PML and RARA domains retained in typical and atypical *PML::RARA* fusions. Since the early death rate in APL patients still reaches 15%, and up to 10% of APL patients are resistant to initial therapy or prone to relapse, further sequencing studies of typical and atypical *PML::RARA* fusion patients might shed light on the pathophysiology of the disease and its responsiveness to treatment.

### 4.3. Additional Gene Fusions Involving Atypical PML::RARA Fusions

For two atypical *PML::RARA* fusions in this study, CENAS also revealed additional genes leading to novel gene fusions.

Case # 13 had a three-way translocation, and the *JOSD1* gene on 22q involved fusions of *PML* and *RARA* genes. JOSD1 belongs to a family of Josephin domain-containing deubiquitinating enzymes [57]. The activity of JOSD1 is regulated by monoubiquitination. JOSD1 plays a role in cytoskeletal dynamics, cell motility, and endocytosis [58]. As a deubiquitinase, JOSD1 is reported to be an ideal therapeutic target and a promising diagnostic marker. JOSD1 is a novel regulator of mutant JAK2 [59]. It interacts with and stabilizes JAK2-V617F, which leads to JAK2-V617F protein degradation due to increased ubiquitination levels. Targeting JOSD1 using small molecule inhibition could be a novel targeted therapy for leukemias with mutant JAK2 [59]. Upregulated JOSD1 contributes to the acquisition of chemo-resistance by inhibiting mitochondrial apoptotic signaling in gynecological cancer by stabilizing MCL1 [60,61]. Depletion of JOSD1 leads to the death of gynecological cancer cells [60]. Further studying the potential pathogenesis of novel *JOSD1* fusions with the *PML* and *RARA* genes in case #13 might be useful for targeted therapy and prediction of chemo-resistance and prognosis.

CENAS revealed that the *ANKFN1* gene on 17q involved fusions of *PML* and *RARA* genes in case #11, likely due to an insertional fusion. Besides its roles in vestibular-related functions [62], *ANKFN1* plays pro-tumorigenic and metastatic roles in hepatocellular carcinoma [63]. It is a recurrent HBV integration event of HBV-infected intrahepatic cholangiocarcinoma [64]. It was found to be a pivotal gene corresponding to the diagnosis of early lung cancer [65]. While the *ANKFN1::RARA* and *ANKFN1::PML* gene fusions found in case #11 may lead to disrupting the *ANKFN1* gene, further studies will be valuable to understand the pathogenesis of these novel fusions in this case.

### 4.4. Complex Rearrangements in a Cryptic PML::RARA Fusion

CENAS revealed a cryptic *PML::RARA* fusion due to a complex fusion/rearrangement (complex insertional) among *PML*, *RARA*, and chromosome 13 in case #12. In addition to a *PML::RARA* fusion, the band of 13q14.13 is the insertion site, which is intergenic and flanks with the *SIAH3* gene (centromeric) and the *ZC3H13* gene (telomeric). These insertional *PML::RARA* fusions led to complex rearrangements, including *PML::*13q14.13 and *RARA::*13q14.13 fusions. While the pathogenesis of these rearrangements is unclear, the positional effect in gene expression is a well-known phenomenon [66,67,68,69]. These rearrangements may have impacts on chromatin structure, interactions with regulatory elements (enhancers, silencers, or insulators), transcriptional clustering, epigenetic modifications, and chromosomal positioning [53,54,55,56]. These rearrangements may lead to disrupting the normal function of the *PML* and *RARA* genes. Loss and overexpression of PML isoforms can inhibit chromosome-based homology-directed repair and affect the efficiency of homologous recombination [70]. Furthermore, these rearrangements may also have impacts on the *ZC3H13* and *SIAH3* genes located at 13q14.13. The *ZC3H13* gene localizes to nuclear speckles and plays a role in mRNA splicing [71]. Its expression has been used to predict prognosis in acute myeloid leukemia [72,73]. The *SIAH3* gene is a member of the SIAH family and is a RING-containing protein that functions as E3 ubiquitin ligases [74]. Understanding the positional effects related to the *PML*, *RARA*, and 13q14.13-flanking genes (*SIAH3* and *ZC3H13*) could shed light on their role in APL and may aid in predicting the prognosis of the patient.

### 4.5. Multiple Fusions Due to Two Isoderivative Chromosomes 17q

Isochromosome 17q is a chromosomal abnormality that leads to the gain of the entire long arm of chromosome 17 and the loss of the entire short arm of chromosome 17, including the *TP53* gene. Isochromosome of the long arm of the derivative chromosome 17, originating from the translocation t(15;17), isoderivative chromosome ider(17)(q10)t(15; 17), has been reported in estimated less than 5% of APL cases, ranging from 0.6% to 4.9% [75,76,77,78]. The presence of two ider(17)(q10)t(15;17) chromosomes results in extra fusion signals by FISH analysis as found in case #14. Its presence is indicative of additional genetic complexity during the formation and development of APL.

While isochromosome 17q is commonly associated with a worse prognosis in hematologic malignancies [79,80], prognostic significance of ider(17)(q10)t(15;17) in APL remains controversial. Given a low incidence of these ider(17)(q10)t(15;17)-positive APL cases, various prognostic significance has been reported. Few ider(17)(q10)t(15;17)-positive APL cases have been reported to follow an unfavorable course and have short survival and poor prognosis [77,78,81,82]. On the other hand, ider(17)(q10)t(15;17)-positive APL cases have been reported to not cause an unfavorable course of APL in the group of patients treated with ATRA and chemotherapy [83]. Therefore, more studies are required to accurately assess the potential clinical impacts of ider(17)(q10)t(15;17) in APL. Currently, the presence of ider(17)(q10)t(15;17) in APL does not change the therapeutic approach and patients are treated with standard ATRA treatment and chemotherapy.

### 4.6. Limitation of CENAS Approach

The amplification-free, sequencing-based CENAS approach is designed to rapidly detect *PML::RARA* fusions in APL patients at initial diagnosis, with a high percentage of APL cancer cells (>20%). It is unsuitable for performing a sensitive and specific quantitative evaluation of minimal residual disease (MRD) during follow-up with a low percentage of APL cancer cells.

## 5. Conclusions

CENAS, an amplification-free nanopore sequencing-based approach with adaptive sampling, is useful for rapidly detecting *PML::RARA* fusions in APL patients. Given its low cost and easy setup, CENAS has the potential for further development as a point-of-care test to offer an immediate bedside diagnosis of APL with a *PML::RARA* fusion. CENAS allowed for the identification of sequence information of fusion breakpoints involved in typical and atypical *PML::RARA* fusions and identified additional genes and genomic regions involving the atypical *PML::RARA* fusions. To the best of our knowledge, involvements of the *ANKFN1* gene, the *JOSD1* gene, and the specific 13q14.13 genomic region have not been reported in the atypical *PML::RARA* fusions. The biological significance of most atypical *PML::RARA* transcripts remains unclear. Thus, there is a need for multi-center studies that combine sequence analysis of atypical *PML::RARA* fusion cases, comprehensive clinical data and genomic data, and uniform treatment strategies. Such studies will shed light on how atypical *PML::RARA* fusions in APL patients are related to treatment response rates, survival, relapse, and overall prognosis. Understanding the involvement of additional genes and positional effects related to the *PML* and *RARA* genes could shed light on their role in APL and may aid in the development of targeted therapies.

## Figures and Tables

**Table 1:** Standard-of-care genetic tests and CENAS-based nanopore sequencing in this study.

Sample ID	FISH (*PML::RARA* Fusion Pattern by Interphase FISH)	Conventional Chromosome Analysis/SNP Microarray	qRT-PCR/the Gene Fusion Assay	Nanopore Sequencing for *PML::RARA* Fusion	
				*PML* Breakpoint	*RARA* Breakpoint
1. APL cell line (NB4)	Positive (atypical, 3R2G2F)	Complex with t(15;17)(q24;q21) and gain of chr15 and chr17	n.a.	Positive, chr15:74326373	Positive, chr17:38502175
2. Non-APL cell *	Negative	Normal	n.a.	Negative	Negative
3: APL	Positive (typical, 1R1G2F)	46,XX,t(8;16)(q24.1; q13),t(15;17)(q24;q21)	n.a.	Positive, chr15:74317057	Positive, chr17:38500768
4: APL	Positive (typical, 1R1G2F)	46,XX,t(15;17)(q24; q21)	*PML::RARA* S transcripts	Positive, chr15:74315881	Positive, chr17:38499739
5: APL	Positive (typical, 1R1G2F)	46,XY,t(15;17)(q24; q21)	*PML::RARA* S transcripts	Positive, chr15:74317119	Positive, chr17:38496479
6: APL	Positive (typical, 1R1G2F)	46,XX,t(15;17)(q24; q21)	*PML::RARA* S transcripts	Positive, chr15:74316942	Positive, chr17:38487124
7: APL	Positive (typical, 1R1G2F)	46,XY,t(15;17)(q24; q21)	*PML::RARA* S transcripts	Positive, chr15:74316541	Positive, chr17:38489466
8: APL	Positive (typical, 1R1G2F)	46,XY,t(15;17)(q24; q21)	*PML::RARA* L transcripts	Positive, chr15:74325390	Positive, chr17:38503907
9: APL	Positive (typical, 1R1G2F)	46,XX,t(15;17)(q24; q21)	*PML::RARA* S transcripts	Positive, chr15:74316942	Positive, chr17:38487124
10: APL	Positive (typical, 1R1G2F)	46,XY,r(6)(p25q23), t(15;17)(q24;q21)	n.a.	Positive, chr15:74326146	Positive, chr17:38500560
11: APL	Positive (atypical, 1R2G1F)	n.a./normal SNP microarray	*PML::RARA* S transcripts	Positive, chr15:74316787	Positive, chr17:38489189
12: APL	Positive (atypical, 1R2G1F)	46,XY,t(13;15)(q14; q24)/normal SNP microarray	Negative	Positive, chr15:74326463	Positive, chr17:38501782
13: APL	Positive (atypical, 2R2G1F)	47,XY,+ 8,t(15;22; 17)(q24;q13;q21)	*PML::RARA* L transcripts	Positive, chr15:74326356	Positive, chr17:38503146
14: APL	Positive (atypical, 1R1G4F−5F)	47,XX,der(15)t(15;17) (q24;q21),−16,+17,ider (17)(q10)t(15;17)(q24; q21)x2,+22	*PML::RARA* L transcripts	Positive, chr15:74326210	Positive, chr17:38501461
15–20 **: Non-APL	Negative	Normal karyotype	Negative	Negative	Negative

* #2 is a non-APL cell line, GM12878; ** #15 to #20 samples are non-APL, obtained from patients with acute myeloid leukemia; APL: acute promyelocytic leukemia; chr: chromosome; L: the long (bcr1) isoform; n.a.: not available; S: the short (bcr3) isoform. Breakpoint sequences aligned to human genome builder GRCh37/hg19.

## Data Availability

All requests for primary data and experimental reagents should be addressed to: yzou19@jhmi.edu.

## Supplementary Materials

The following supporting information can be downloaded at: https://www.mdpi.com/article/10.3390/biom14121595/s1, Figure S1: *PML/RARA* FISH in NB4 and GM12878. Figure S2: *PML::RARA* gene fusion by the NGS sequencing. Table S1: Characteristics of the CENAS runs and enrichment.

## Abbreviations

AML	Acute myeloid leukemia
ANKFN1	Ankyrin repeat- and fibronectin type III domain-containing protein 1
APL	Acute promyelocytic leukemia
Cas9	CRISPR-associated Protein 9
CENAS	CRISPR/Cas9-enriched nanopore sequencing with adaptive sampling
CNV	Copy number variants
CRISPR	Clustered regularly interspaced short palindromic repeats
FISH	Fluorescence in situ hybridization
guideRNA	Guide ribonucleic acid ider isoderivative
IDT	Integrated DNA Technologies
IGV	Integrative Genomics Viewer
*JOSD1*	Josephin domain-containing protein 1
MDS	Myelodysplastic syndrome
NGS	Next-generation sequencing
*PML*	Promyelocytic leukemia gene
*RARA*	Retinoic acid receptor alpha gene
*SIAH3*	SIAH E3 ubiquitin protein ligase family, member 3
SVs	Structural variants
*ZC3H13*	Zinc finger CCCH domain-containing protein 13

## References

[1] Grimwade D., Lo Coco F. (2002). Acute promyelocytic leukemia: A model for the role of molecular diagnosis and residual disease monitoring in directing treatment approach in acute myeloid leukemia. Leukemia.

[2] Sanz M.A., Fenaux P., Tallman M.S., Estey E.H., Lowenberg B., Naoe T., Lengfelder E., Dohner H., Burnett A.K., Chen S. (2019). Management of acute promyelocytic leukemia: Updated recommendations from an expert panel of the European LeukemiaNet. Blood.

[3] Liquori A., Ibanez M., Sargas C., Sanz M.A., Barragan E., Cervera J. (2020). Acute Promyelocytic Leukemia: A Constellation of Molecular Events around a Single *PML-RARA* Fusion Gene. Cancers.

[4] Park T.S., Kim J.S., Song J., Lee K.A., Yoon S., Suh B., Lee J., Lee H., Kim J., Choi J. (2009). Acute promyelocytic leukemia with insertion of *PML* exon 7a and partial deletion of exon 3 of *RARA*: A novel variant transcript related to aggressive course and not detected with real-time polymerase chain reaction analysis. Cancer Genet. Cytogenet..

[5] Iaccarino L., Divona M., Ottone T., Cicconi L., Lavorgna S., Ciardi C., Alfonso V., Travaglini S., Facchini L., Cimino G. (2019). Identification and monitoring of atypical *PML/RARA* fusion transcripts in acute promyelocytic leukemia. Genes Chromosomes Cancer.

[6] Jeziskova I., Razga F., Gazdova J., Doubek M., Jurcek T., Koristek Z., Mayer J., Dvoráková D. (2010). A case of a novel *PML/RARA* short fusion transcript with truncated transcription variant 2 of the *RARA* gene. Mol. Diagn. Ther..

[7] Walz C., Grimwade D., Saussele S., Lengfelder E., Haferlach C., Schnittger S., Lafage-Pochitaloff M., Hochhaus A., Cross N.C.P., Reiter A. (2010). Atypical mRNA fusions in *PML-RARA* positive, *RARA-PML* negative acute promyelocytic leukemia. Genes Chromosomes Cancer.

[8] McKinney C.D., Golden W.L., Gemma N.W., Swerdlow S.H., Williams M.E. (1994). *RARA* and *PML* gene rearrangements in acute promyelocytic leukemia with complex translocations and atypical features. Genes Chromosomes Cancer..

[9] Bartalucci N., Romagnoli S., Vannucchi A.M. (2022). A blood drop through the pore: Nanopore sequencing in hematology. Trends Genet..

[10] Avershina E., Frye S.A., Ali J., Taxt A.M., Ahmad R. (2022). Ultrafast and Cost-Effective Pathogen Identification and Resistance Gene Detection in a Clinical Setting Using Nanopore Flongle Sequencing. Front. Microbiol..

[11] Gradel C., Terrazos Miani M.A., Barbani M.T., Leib S.L., Suter-Riniker F., Ramette A. (2019). Rapid and Cost-Efficient Enterovirus Genotyping from Clinical Samples Using Flongle Flow Cells. Genes.

[12] Loose M., Malla S., Stout M. (2016). Real-time selective sequencing using nanopore technology. Nat. Methods.

[13] Payne A., Holmes N., Rakyan V., Loose M. (2019). BulkVis: A graphical viewer for Oxford nanopore bulk FAST5 files. Bioinformatics.

[14] Kovaka S., Fan Y., Ni B., Timp W., Schatz M.C. (2021). Targeted nanopore sequencing by real-time mapping of raw electrical signal with UNCALLED. Nat. Biotechnol..

[15] Edwards H.S., Krishnakumar R., Sinha A., Bird S.W., Patel K.D., Bartsch M.S. (2019). Real-Time Selective Sequencing with RUBRIC: Read Until with Basecall and Reference-Informed Criteria. Sci. Rep..

[16] Weilguny L., De Maio N., Munro R., Manser C., Birney E., Loose M., Goldman N. (2023). Dynamic, adaptive sampling during nanopore sequencing using Bayesian experimental design. Nat. Biotechnol..

[17] Naarmann-de Vries I.S., Gjerga E., Gandor C.L.A., Dieterich C. (2023). Adaptive sampling for nanopore direct RNA-sequencing. RNA.

[18] Jinek M., Chylinski K., Fonfara I., Hauer M., Doudna J.A., Charpentier E. (2012). A programmable dual-RNA-guided DNA endonuclease in adaptive bacterial immunity. Science.

[19] Hsu P.D., Lander E.S., Zhang F. (2014). Development and applications of CRISPR-Cas9 for genome engineering. Cell.

[20] Bak R.O., Dever D.P., Porteus M.H. (2018). CRISPR/Cas9 genome editing in human hematopoietic stem cells. Nat. Protoc..

[21] Hendel A., Bak R.O., Clark J.T., Kennedy A.B., Ryan D.E., Roy S., Steinfeld I., Lunstad B.D., Kaiser R.J., Wilkens A.B. (2015). Chemically modified guide RNAs enhance CRISPR-Cas genome editing in human primary cells. Nat. Biotechnol..

[22] Dasgupta I., Flotte T.R., Keeler A.M. (2021). CRISPR/Cas-Dependent and Nuclease-Free *In Vivo* Therapeutic Gene Editing. Hum. Gene Ther..

[23] Wu Y., Zeng J., Roscoe B.P., Liu P., Yao Q., Lazzarotto C.R., Clement K., Cole M.A., Luk K., Baricordi C. (2019). Highly efficient therapeutic gene editing of human hematopoietic stem cells. Nat. Med..

[24] Canver M.C., Smith E.C., Sher F., Pinello L., Sanjana N.E., Shalem O., Chen D.D., Schupp P.G., Vinjamur D.S., Garcia S.P. (2015). *BCL11A* enhancer dissection by Cas9-mediated in situ saturating mutagenesis. Nature.

[25] Gillmore J.D., Gane E., Taubel J., Kao J., Fontana M., Maitland M.L., Seitzer J., O’Connell D., Walsh K.R., Wood K. (2021). CRISPR-Cas9 In Vivo Gene Editing for Transthyretin Amyloidosis. N. Engl. J. Med..

[26] Li H., Yang Y., Hong W., Huang M., Wu M., Zhao X. (2020). Applications of genome editing technology in the targeted therapy of human diseases: Mechanisms, advances and prospects. Signal Transduct. Target. Ther..

[27] Kim J.S. (2016). Genome editing comes of age. Nat. Protoc..

[28] Strong A., Musunuru K. (2017). Genome editing in cardiovascular diseases. Nat. Rev. Cardiol..

[29] Frangoul H., Altshuler D., Cappellini M.D., Chen Y.S., Domm J., Eustace B.K., Foell J., de la Fuente J., Grupp S., Handgretinger R. (2021). CRISPR-Cas9 Gene Editing for Sickle Cell Disease and beta-Thalassemia. N. Engl. J. Med..

[30] Sharma A., Boelens J.J., Cancio M., Hankins J.S., Bhad P., Azizy M., Lewandowski A., Zhao X., Chitnis S., Peddinti R. (2023). CRISPR-Cas9 Editing of the *HBG1* and *HBG2* Promoters to Treat Sickle Cell Disease. N. Engl. J. Med..

[31] Longhurst H.J., Lindsay K., Petersen R.S., Fijen L.M., Gurugama P., Maag D., Butler J.S., Shah M.Y., Golden A., Xu Y. (2024). CRISPR-Cas9 In Vivo Gene Editing of *KLKB1* for Hereditary Angioedema. N. Engl. J. Med..

[32] Gilpatrick T., Lee I., Graham J.E., Raimondeau E., Bowen R., Heron A., Downs B., Sukumar S., Sedlazeck F.J., Timp W. (2020). Targeted nanopore sequencing with Cas9-guided adapter ligation. Nat. Biotechnol..

[33] Phan M., Gomes M.A., Stinnett V., Morsberger L., Hoppman N.L., Pearce K.E., Smith K., Phan B., Jiang L., Zou Y.S. (2024). An Integrated Approach Including CRISPR/Cas9-Mediated Nanopore Sequencing, Mate Pair Sequencing, and Cytogenomic Methods to Characterize Complex Structural Rearrangements in Acute Myeloid Leukemia. Biomedicines.

[34] McGowan-Jordan J.H.R., Moore  S. (2020). ISCN 2020: An International System for Human Cytogenomic Nomenclature.

[35] Jiang L., Pallavajjala A., Huang J., Haley L., Morsberger L., Stinnett V., Hardy M., Park R., Ament C., Finch A. (2021). Clinical Utility of Targeted Next-Generation Sequencing Assay to Detect Copy Number Variants Associated with Myelodysplastic Syndrome in Myeloid Malignancies. J. Mol. Diagn..

[36] Haley L., Parimi V., Jiang L., Pallavajjala A., Hardy M., Yonescu R., Morsberger L., Stinnett V., Long P., Zou Y.S. (2021). Diagnostic Utility of Gene Fusion Panel to Detect Gene Fusions in Fresh and Formalin-Fixed, Paraffin-Embedded Cancer Specimens. J. Mol. Diagn..

[37] Pallavajjala A., Haley L., Stinnett V., Adams E., Pallavajjala R., Huang J., Morsberger L., Hardy M., Long P., Gocke C.D. (2022). Utility of targeted next-generation sequencing assay to detect 1p/19q co-deletion in formalin-fixed paraffin-embedded glioma specimens. Hum. Pathol..

[38] Grimwade D., Biondi A., Mozziconacci M.J., Hagemeijer A., Berger R., Neat M., Howe K., Dastugue N., Jansen J., Radford-Weiss I. (2000). Characterization of acute promyelocytic leukemia cases lacking the classic t(15;17): Results of the European Working Party. Groupe Francais de Cytogenetique Hematologique, Groupe de Francais d’Hematologie Cellulaire, UK Cancer Cytogenetics Group and BIOMED 1 European Community-Concerted Action “Molecular Cytogenetic Diagnosis in Haematological Malignancies”. Blood.

[39] Gagnon M.F., Berg H.E., Meyer R.G., Sukov W.R., Van Dyke D.L., Jenkins R.B., Greipp P.T., Thorland E.C., Hoppman N.L., Xu X. (2022). Typical, atypical and cryptic t(15;17)(q24;q21) (*PML::RARA*) observed in acute promyelocytic leukemia: A retrospective review of 831 patients with concurrent chromosome and *PML::RARA* dual-color dual-fusion FISH studies. Genes Chromosomes Cancer.

[40] Sereika M., Kirkegaard R.H., Karst S.M., Michaelsen T.Y., Sorensen E.A., Wollenberg R.D., Albertsen M. (2022). Oxford Nanopore R10.4 long-read sequencing enables the generation of near-finished bacterial genomes from pure cultures and metagenomes without short-read or reference polishing. Nat. Methods.

[41] Linde J., Brangsch H., Holzer M., Thomas C., Elschner M.C., Melzer F., Tomaso H. (2023). Comparison of Illumina and Oxford Nanopore Technology for genome analysis of *Francisella tularensis*, *Bacillus anthracis*, and *Brucella suis*. BMC Genom..

[42] Sanderson N.D., Hopkins K.M.V., Colpus M., Parker M., Lipworth S., Crook D., Stoesser N. (2024). Evaluation of the accuracy of bacterial genome reconstruction with Oxford Nanopore R10.4.1 long-read-only sequencing. Microb. Genom..

[43] Zhao W., Zeng W., Pang B., Luo M., Peng Y., Xu J., Kan B., Li Z., Lu X. (2023). Oxford nanopore long-read sequencing enables the generation of complete bacterial and plasmid genomes without short-read sequencing. Front. Microbiol..

[44] Wick R.R., Judd L.M., Holt K.E. (2023). Assembling the perfect bacterial genome using Oxford Nanopore and Illumina sequencing. PLoS Comput. Biol..

[45] Delahaye C., Nicolas J. (2021). Sequencing DNA with nanopores: Troubles and biases. PLoS ONE.

[46] Zhang X., Liu C.G., Yang S.H., Wang X., Bai F.W., Wang Z. (2022). Benchmarking of long-read sequencing, assemblers and polishers for yeast genome. Brief. Bioinform..

[47] Ramani N.S., Patel K.P., Routbort M.J., Alvarez H., Broaddus R., Chen H., Rashid A., Lazar A., San Lucas F.A., Yao H. (2021). Factors Impacting Clinically Relevant RNA Fusion Assays Using Next-Generation Sequencing. Arch. Pathol. Lab. Med..

[48] Zheng Z., Liebers M., Zhelyazkova B., Cao Y., Panditi D., Lynch K.D., Chen J., Robinson H.E., Shim H.S., Chmielecki J. (2014). Anchored multiplex PCR for targeted next-generation sequencing. Nat. Med..

[49] Klausner M., Stinnett V., Ghabrial J., Morsberger L., DeMetrick N., Long P., Zhu J., Smith K., James T., Adams E. (2024). Optical Genome Mapping Reveals Complex and Cryptic Rearrangement Involving *PML::RARA* Fusion in Acute Promyelocytic Leukemia. Genes.

[50] Cao Y., Yao L., Liu Y., Gu Q., Dong W., Wang Z., Wang F., Lin R., Xie X., Cen J. (2019). An Atypical *PML-RARA* Rearrangement Resulting from Submicroscopic Insertion of the *RARA* Gene at the *PML* Locus with Novel Breakpoints within *PML* Exon 7b and *RARA* Exon 3. Acta Haematol..

[51] Lauricella C., Greco R., Mancini V., Motta V., Ciraolo A., De Canal G., De Paoli E., Paglino G., Guido V., Bonoldi E. (2023). Acute Promyelocytic Leukemia with del(6)(p22) and Atypical bcr2 *PML::RARA* Fusion Transcript: A Case Report. Acta Haematol..

[52] Ismail S., Ababneh N., Awidi A. (2007). Identification of atypical *PML-RARA* breakpoint in a patient with acute promyelocytic leukemia. Acta Haematol..

[53] Gallagher R.E., Li Y.P., Rao S., Paietta E., Andersen J., Etkind P., Bennett J.M., Tallman M.S., Wiernik P.H. (1995). Characterization of acute promyelocytic leukemia cases with *PML-RAR* alpha break/fusion sites in *PML* exon 6: Identification of a subgroup with decreased in vitro responsiveness to all-trans retinoic acid. Blood.

[54] Gu B.W., Xiong H., Zhou Y., Chen B., Wang L., Dong S., Yu Z., Lu L., Zhong M., Yin H. (2002). Variant-type PML-RARα fusion transcript in acute promyelocytic leukemia: Use of a cryptic coding sequence from intron 2 of the *RAR*α gene and identification of a new clinical subtype resistant to retinoic acid therapy. Proc. Natl. Acad. Sci. USA.

[55] Slack J.L., Willman C.L., Andersen J.W., Li Y.P., Viswanatha D.S., Bloomfield C.D., Tallman M.S., Gallagher R.E. (2000). Molecular analysis and clinical outcome of adult APL patients with the type V PML-RARα isoform: Results from intergroup protocol 0129. Blood.

[56] Chillon M.C., Gonzalez M., Garcia-Sanz R., Balanzategui A., Gonzalez D., Lopez-Perez R., Mateos M.V., Alaejos I., Rayón C., Arbeteta J. (2000). Two new 3’ *PML* breakpoints in t(15;17)(q22;q21)-positive acute promyelocytic leukemia. Genes Chromosomes Cancer.

[57] Nomura N., Nagase T., Miyajima N., Sazuka T., Tanaka A., Sato S., Seki N., Kawarabayasi Y., Ishikawa K., Tabata S. (1994). Prediction of the coding sequences of unidentified human genes, I.I. The coding sequences of 40 new genes (KIAA0041-KIAA0080) deduced by analysis of cDNA clones from human cell line KG-1. DNA Res..

[58] Seki T., Gong L., Williams A.J., Sakai N., Todi S.V., Paulson H.L. (2013). JosD1, a membrane-targeted deubiquitinating enzyme, is activated by ubiquitination and regulates membrane dynamics, cell motility, and endocytosis. J. Biol. Chem..

[59] Yang J., Weisberg E.L., Liu X., Magin R.S., Chan W.C., Hu B., Schauer N.J., Zhang S., Ilaria Lamberto I., Doherty L. (2022). Small molecule inhibition of deubiquitinating enzyme JOSD1 as a novel targeted therapy for leukemias with mutant JAK2. Leukemia.

[60] Wu X., Luo Q., Zhao P., Chang W., Wang Y., Shu T., Ding F., Li B., Liu Z. (2020). JOSD1 inhibits mitochondrial apoptotic signalling to drive acquired chemoresistance in gynaecological cancer by stabilizing MCL1. Cell Death Differ..

[61] Li M., Gao F., Li X., Gan Y., Han S., Yu X., Liu H., Li W. (2022). Stabilization of MCL-1 by E3 ligase TRAF4 confers radioresistance. Cell Death Dis..

[62] Ross K.D., Ren J., Zhang R., Chi N.C., Hamilton B.A. (2022). *Ankfn1*-mutant vestibular defects require loss of both ancestral and derived paralogs for penetrance in zebrafish. G3.

[63] Wang Y., Zhang Y., Mi J., Jiang C., Wang Q., Li X., Zhao M., Geng Z., Song X., Li J. (2022). ANKFN1 plays both protumorigenic and metastatic roles in hepatocellular carcinoma. Oncogene.

[64] Zhao L., Wang Y., Tian T., Rao X., Dong W., Zhang J., Yang Y., Tao Q., Peng F., Shen C. (2022). Analysis of viral integration reveals new insights of oncogenic mechanism in HBV-infected intrahepatic cholangiocarcinoma and combined hepatocellular-cholangiocarcinoma. Hepatol. Int..

[65] Qureshi N., Chi J., Qian Y., Huang Q., Duan S. (2022). Looking for the Genes Related to Lung Cancer from Nasal Epithelial Cells by Network and Pathway Analysis. Front. Genet..

[66] Quina A.S., Parreira L. (2005). Telomere-surrounding regions are transcription-permissive 3D nuclear compartments in human cells. Exp. Cell Res..

[67] Baur J.A., Zou Y., Shay J.W., Wright W.E. (2001). Telomere position effect in human cells. Science.

[68] Spielmann M., Lupianez D.G., Mundlos S. (2018). Structural variation in the 3D genome. Nat. Rev. Genet..

[69] Shaul O. (2017). How introns enhance gene expression. Int. J. Biochem. Cell Biol..

[70] Attwood K.M., Salsman J., Chung D., Mathavarajah S., Van Iderstine C., Dellaire G. (2020). PML isoform expression and DNA break location relative to PML nuclear bodies impacts the efficiency of homologous recombination. Biochem. Cell Biol..

[71] Horiuchi K., Kawamura T., Iwanari H., Ohashi R., Naito M., Kodama T., Hamakubo T. (2013). Identification of Wilms’ tumor 1-associating protein complex and its role in alternative splicing and the cell cycle. J. Biol. Chem..

[72] Liao X., Chen L., Liu J., Hu H., Hou D., You R., Wang X., Huang H. (2023). m^6^A RNA methylation regulators predict prognosis and indicate characteristics of tumour microenvironment infiltration in acute myeloid leukaemia. Epigenetics.

[73] Xu J.R., Yang D.H., Long G.F., Sun H., Chen H.B. (2021). [Establishment and validation of prognosis predictive model using m^6^A RNA methylation regulators in children acute myeloid leukemia]. Zhonghua Yu Fang. Yi Xue Za Zhi.

[74] Robbins C.M., Tembe W.A., Baker A., Sinari S., Moses T.Y., Beckstrom-Sternberg S., Beckstrom-Sternberg J., Barrett M., Long J., Chinnaiyan A. (2011). Copy number and targeted mutational analysis reveals novel somatic events in metastatic prostate tumors. Genome Res..

[75] Gohring G., Lange K., Atta J., Krauter J., Holzer D., Schlegelberger B. (2007). Cryptic t(15;17) in a patient with AML M3 and a complex karyotype. Cancer Genet. Cytogenet..

[76] De Botton S., Chevret S., Sanz M., Dombret H., Thomas X., Guerci A., Fey M., Rayon C., Huguet F., Sotto J.J. (2000). Additional chromosomal abnormalities in patients with acute promyelocytic leukaemia (APL) do not confer poor prognosis: Results of APL 93 trial. Br. J. Haematol..

[77] Kim M.J., Yoon H.S., Cho S.Y., Lee H.J., Suh J.T., Lee J., Yoon H., Lee W., Park T.S. (2010). ider(17)(q10)t(15;17) associated with relapse and poor prognosis in a pediatric patient with acute promyelocytic leukemia. Cancer Genet. Cytogenet..

[78] Tong H., Li K., Mei C., Wang H., Chen Z., Jin J. (2011). Arsenic trioxide may improve the prognosis of APL with ider(17)(q10): Report of a rare adult case of acute promyelocytic leukemia with ider(17)(q10)t(15;17) showing poor response to all-trans retinoic acid. Ann. Hematol..

[79] Campo E., Cymbalista F., Ghia P., Jager U., Pospisilova S., Rosenquist R., Schuh A., Stilgenbauer S. (2018). *TP53* aberrations in chronic lymphocytic leukemia: An overview of the clinical implications of improved diagnostics. Haematologica.

[80] Middeke J.M., Fang M., Cornelissen J.J., Mohr B., Appelbaum F.R., Stadler M., Sanz J., Baurmann H., Bug G., Schäfer-Eckart K. (2014). Outcome of patients with abnl(17p) acute myeloid leukemia after allogeneic hematopoietic stem cell transplantation. Blood.

[81] Cervera J., Montesinos P., Hernandez-Rivas J.M., Calasanz M.J., Aventin A., Ferro M.T., Luño E., Sánchez J., Vellenga E., Rayón C. (2010). Additional chromosome abnormalities in patients with acute promyelocytic leukemia treated with all-trans retinoic acid and chemotherapy. Haematologica.

[82] Lee G.Y., Christina S., Tien S.L., Ghafar A.B., Hwang W., Lim L.C., Lim T.H. (2005). Acute promyelocytic leukemia with *PML-RARA* fusion on i(17q) and therapy-related acute myeloid leukemia. Cancer Genet. Cytogenet..

[83] Manola K.N., Karakosta M., Sambani C., Terzoudi G., Pagoni M., Gatsa E., Papaioannou M. (2010). Isochromosome der(17)(q10)t(15;17) in acute promyelocytic leukemia resulting in an additional copy of the *RARA-PML* fusion gene: Report of 4 cases and review of the literature. Acta Haematol..

