# Supplementary material for: Rapid Detection of PML::RARA Fusions in Acute Promyelocytic Leukemia: CRISPR/Cas9 Nanopore Sequencing with Adaptive Sampling
Source: Biomolecules. 2024 Dec 13;14(12):1595. doi: 10.3390/biom14121595 (PMC11674480; doi:10.3390/biom14121595)
Supplement: Supplementary file 1 [file biomolecules-14-01595-s001.zip › biomolecules-3295666-supplementary.pdf]

biomolecules

Supplementary materials

Rapid Detection of *PML::RARA* Fusions in Acute Promyelocytic Leukemia: CRISPR/Cas9 Nanopore Sequencing with Adaptive Sampling

William Middlezong [1], Victoria Stinnett [2], Michael Phan [1], Brian Phan [3], Laura Morsberger [2], Melanie Klausner [2], Jen Ghabrial [2], Natalie DeMetrick [2], Jing Zhu [2], Trisha James [2], Aparna Pallavajjala [2], Christopher D. Gocke [2], Maria R. Baer [4] and Ying S. Zou [2,*]

[1] Krieger School of Arts and Sciences, Johns Hopkins University, Baltimore, MD21218, USA; wmiddle2@jhu.edu (W.M.); mphan4@jhu.edu (M.P.);
[2] Department of Pathology, Johns Hopkins University School of Medicine, Baltimore, MD21287, USA; lmorsber@jhmi.edu (L.M.); mhardy22@jhmi.edu (M.K.), cgocke1@jhmi.edu (C.D.G.), vlloyd3@jhmi.edu (V.S.), jghabri1@jh.edu (J.G.), nescola1@jhu.edu (N.D.), jzhu23@jh.edu (J.Z.), tjames17@jhmi.edu (T.J.), apallav2@jhmi.edu (A.P.);
[3] Department of Biology, The College of William and Mary, Williamsburg, VA23186, USA; bphan3@jh.edu (B.P.),
[4] Department of Medicine, University of Maryland Greenebaum Comprehensive Cancer Center, Baltimore, MD21201, USA; mbaer@umm.edu (M.R.B.);
* Correspondence: yzou19@jhmi.edu

Table S1. Characteristics of the CENAS runs and enrichment.

Sample Name	Sample type	Sequence Device	Flow cell	MB of Reads	Mean Coverage	All Reads	Target Reads *	*PML* Depth	*PML* Enrichment	*RARA* Depth	*RARA* Enrichment	Median PHRED	Reads > Q10	Run time (hr.)
NB4	Cell line	Mk1b	Flongle	5.3	0.0018	2246	26	0.33	187×	0.66	374×	7	255	24
APL-1	Blood	Mk1b	MinION	656.1	0.2187	410199	8748	153.50	701×	253.76	1160×	11.5	323974	12
APL-2	Bone marrow	Mk1b	Flongle	2.8	0.0009	654	8	0.90	962×	0.59	631×	10.2	355	24
APL-3	Bone marrow	GridION	Flongle	1.7	0.0006	1283	12	0.45	797×	0.26	460×	9.8	574	24
APL-4	Blood	Mk1b	MinION	35.9	0.0120	30848	429	10.04	839×	10.13	847×	12.1	25933	2
APL-5	Bone marrow	Mk1b	Flongle	0.8	0.0003	1244	9	0.10	383×	0.20	732×	6.8	259	24
APL-6	Bone marrow	GridION	Flongle	1.1	0.0004	1250	12	0.26	703×	0.27	732×	4.9	350	24
APL-7	Blood	GridION	Flongle	1.1	0.0004	2207	6	0.08	227×	0.08	221×	4.9	115	24

APL-8	Bone marrow	GridION	Flongle	2.1	0.0007	8977	8	0.43	614×	0.58	831×	5	542	24
APL-9	Blood	Mk1b	MinION	101.4	0.0338	55330	1178	18.70	553×	18.70	553×	12.1	46573	12
APL-10	Bone marrow	GridION	MinION	991	0.3303	977174	6568	156.84	475×	138.46	419×	11.9	798115	12
APL-11	Blood	Mk1b	MinION	382	0.1273	266087	5315	116.22	913×	151.14	1187×	11.8	210508	12
APL-12	Bone marrow	Mk1b	MinION	178.4	0.0595	101349	3083	55.06	926×	82.47	1387×	11.3	75624	12
Non-APL (#15-20)	Bone marrow	Mk1b	Flongle	1.5-5.2	0.0005–0.0017	1321 – 5501	8 – 12	0.31 – 1.01	187× – 620×	0.31 – 0.62	346×-675x	5.9 – 7.1	245-501	24

APL: acute promyelocytic leukemia; MB: megabase (1 million base pairs); Mk1b: the MinION Mk1b; *: Target reads of *PML* and *RARA*

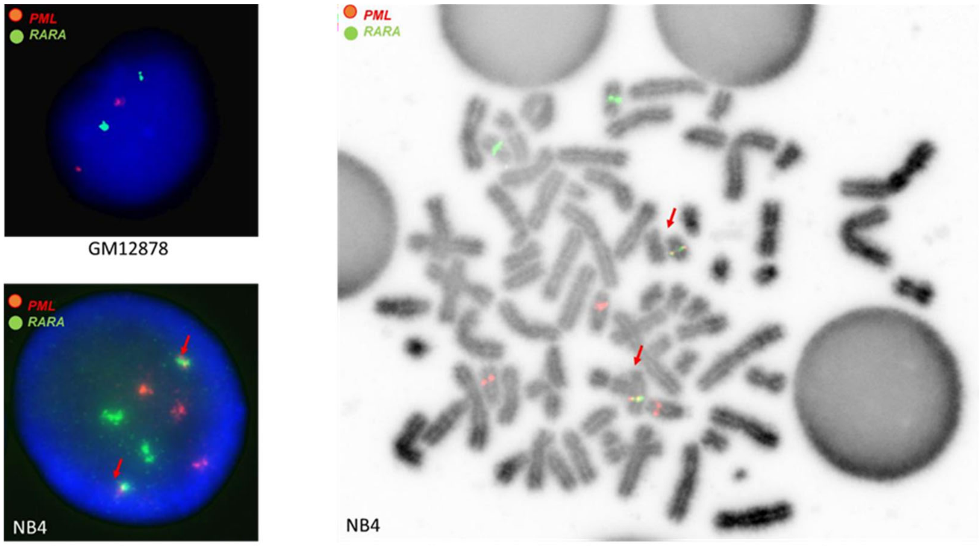

Figure S1. *PML*/*RARA* FISH in NB$ and GM12878. The red arrows point to *PML::RARA* fusions.

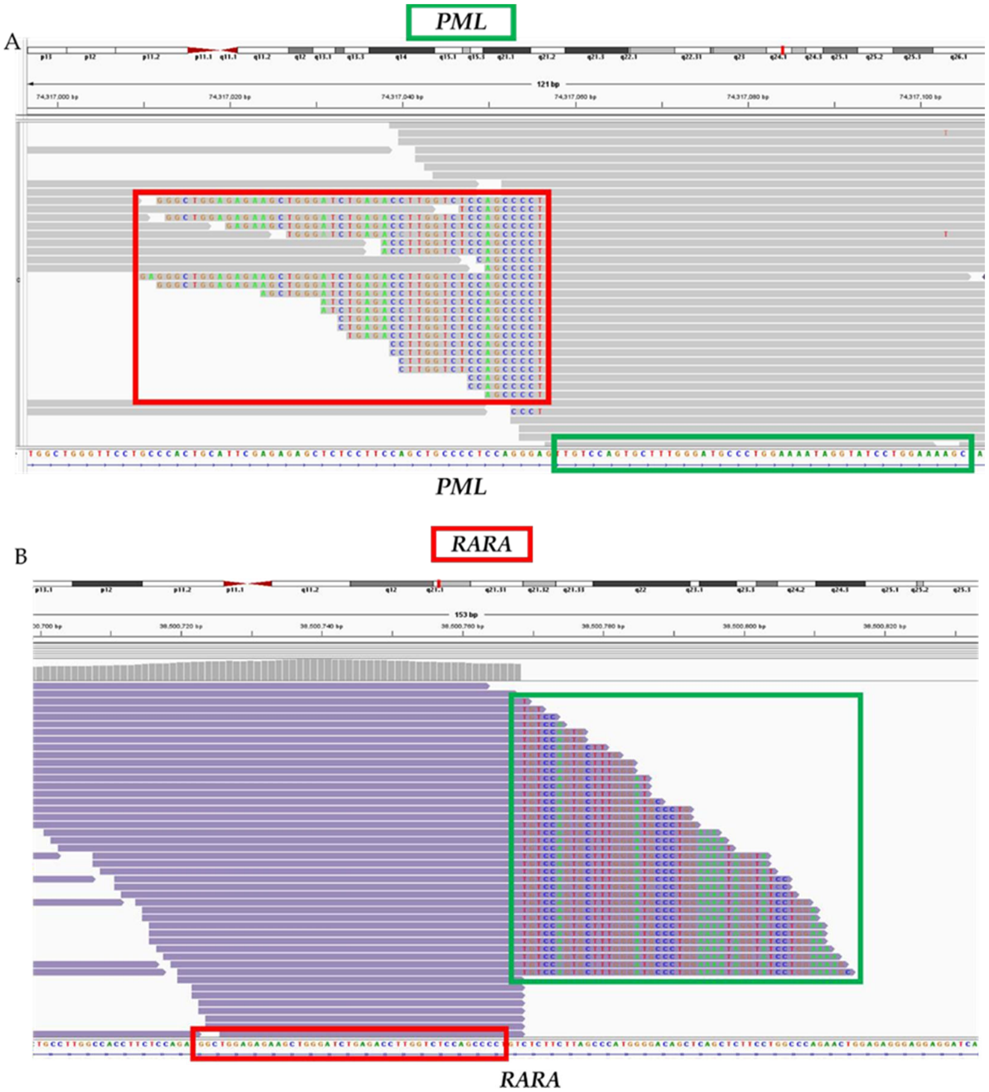

Figure S2. *PML*::*RARA* gene fusion by the NGS sequencing: The DNA sequencing revealed a *PML*::*RARA* gene fusion in case # 3 (Table 1). (**A**). The breakpoint of the *PML* gene was at intron 3 [chromosome (chr), chr15:74317057] shown by green boxes, and (**B**). The breakpoint of the *RARA* gene was at intron 2 (chr17:38500768), shown by red boxes. All analyses were performed using human reference genome assembly GRCh37/hg19.
